# Brand audit attribution of plastic litter in the African Great Lakes: dominance of national manufacturers and implications for extended producer responsibility

**DOI:** 10.1007/s11356-026-38019-w

**Published:** 2026-07-10

**Authors:** Farhan R. Khan, Mamlo A. Yusuph, Christina Sørensen, Bahati S. Mayoma

**Affiliations:** 1 NORCE Norwegian, Research Centre AS, Nygårdsporten 112, NO-5008 Bergen, Norway; 2 Environmental Conservation Community of Tanzania, Mbezi Beach, P.O Box 70132, Dar Es Salaam, Tanzania; 3 Norwegian Institute for Water Research (NIVA), Thormøhlensgate 53D, NO-5006 Bergen, Norway; 4 School of Aquatic Sciences and Fisheries Technology, University of Dar Es Salaam, P.O Box 60091, Dar Es Salaam, Tanzania

**Keywords:** Plastic macro-litter, Anthropogenic litter, Single-use plastics, Corporate responsibility, Waste management policy, East Africa, Tanzania, Freshwater pollution

## Abstract

Plastic pollution is an established environmental crisis, but the responsibility of specific producers remains poorly understood particularly in freshwater systems. The present study applies brand audit–based attribution to identify the manufacturers associated with branded plastic litter collected during a large-scale citizen science clean-up campaign across the Tanzanian shorelines of the African Great Lakes (Lakes Victoria, Tanganyika, and Nyasa (Malawi), A dataset of 11,207 litter items with visible and validated branding was dominated by plastics (9951 items, 88.8%), mainly comprised of single use “fast-moving consumer goods”. Beverage bottles constituted the largest plastic item category (64.9%), followed by soft packaging (1379 items, 13.9%) and food wrappers (1242 items, 12.5%). In total, 292 brands and 148 manufacturers were identified. Corporate contributions were highly concentrated, with three manufacturers (Jambo Group of Companies, Bakhresa Food Products Ltd, and MeTL Group) accounting for 52.9% of branded plastic litter, while 29 manufacturers explained 95.1%. Overall, 91.4% of branded plastic litter was attributable to Tanzanian manufacturers, with regional and international manufacturers representing minor fractions. This pronounced concentration and national embedding of responsibility suggest that Extended Producer Responsibility (EPR) frameworks may be particularly feasible in this context. Coordinated and transparent action to finance waste infrastructure and implement EPR mechanisms, such as bottle return schemes, would send a clear and credible signal of commitment to pollution mitigation. Whilst community-led clean-ups have an important role, without parallel producer responsibility measures and infrastructure development, the burden of waste management continues to fall disproportionately on local communities.

**Supplementary Information:**

The online version contains supplementary material available at 10.1007/s11356-026-38019-w.

## Introduction

Plastic pollution is a ubiquitous global concern connecting across the triple planetary crisis of pollution, climate change, and biodiversity loss (Schmidt et al. 2024), as well as driving the transgression of Earth system boundaries beyond their safe operating spaces (Villarrubia-Gómez et al. 2024). Although the complexity of the “wicked” plastic problem across the life cycle should not be underestimated (Wagner 2021), it can be simplified as the dual pressures of continuously increasing levels of plastic production and consumption, coupled with the ongoing mismanagement of waste (Kibria et al. 2023). While early research focused predominantly on marine environments, plastic pollution in freshwater systems is now well established, with rivers and lakes acting both as major accumulation zones and as critical transport pathways for plastic litter (Guo et al. 2024). Shorelines of rivers and lakes, in particular, act as sinks for plastic litter, reflecting land-based consumption patterns and local waste management effectiveness (van Emmerik et al. 2022). Clean-up campaigns have increasingly contributed to documenting the scale, composition, and spatial distribution of anthropogenic litter, in general, while also confirming the dominance of plastics as the primary material of interest (Nelms et al. 2017; Mayoma et al. 2019; McGoran et al. 2023). In addition to the aesthetic benefits of clean-ups, initiatives based on citizen science and voluntary participation provide further benefits by raising public awareness and engagement in environmental stewardship (Syberg et al. 2018; Nelms et al. 2022). The challenge that persists in addressing plastic pollution is identifying sources and producers, and thus by extension accountability.

A landmark study by Cowger et al. (2024) compiled brand audit data from over fifteen hundred clean-up events across eighty-four countries and several continents showing that, at the global scale, plastic pollution is highly concentrated among a relatively small number of producers. International manufacturers of products termed “fast-moving consumer goods” including foods, beverages and tobacco that rely heavily on single-use plastics, accounted for a quarter of the total number of branded items. The Coca-Cola Company (11%), PepsiCo (5%), Nestlé (3%), Danone (3%), and Altria (2%) were the top five manufacturers, while fifty percent of all branded plastic litter was attributable to just fifty-six companies (Cowger et al. 2024). Such global datasets are influenced by several factors, including the predominance of coastal clean-up events and disparities in waste management infrastructures across the world. Consequently, regional studies may differ from this global analysis and within individual countries suggest that locally branded plastic litter may dominate over multinational producers. For instance, in parts of a Canadian survey of single-use plastic local brands such as Naya, Eska and Sobeys were top litter brands (Baxter et al. 2022) and similarly on the beaches of Sri Lanka domestic packaging material was the main source of beached marine debris (Jang et al. 2018).

Our previous study detailed the outputs of the Clean Shores, Great Lakes clean-up campaign (Mayoma et al. 2025) in which sixty-nine clean-ups were coordinated across the Tanzanian shorelines of the three African Great Lakes, engaging with over 5,000 volunteers. A total of over 430,000 items (almost 26,000 kg) of anthropogenic litter were collected, and although litter density varied with anthropogenic activity across sites, plastic waste was the most prevalent litter type at each location and constituted 75% of all litter. Plastic beverage bottles and plastic bags were the two most abundant litter items, between them constituting 40% of all litter. Beyond clean-ups the project’s holistic approach involved citizen science training, school visits to increase public awareness, and dissemination to relevant policymakers and stakeholders to motivate action on environmental management and the reduction of litter (Mayoma et al. 2025). Building on this previous compositional and spatial analysis, the present study applies brand audit–based attribution to the plastic litter collected during the same clean-ups to identify contributing brands and manufacturers. The main objective of this study is to determine the dominant brands and manufacturers contributing to branded plastic litter in the Tanzanian African Great Lakes and assess the concentration of corporate contributions. By aggregating branded items to the manufacturer level, we assess the role of corporate contributions to plastic litter specific to the African Great Lakes. The findings provide evidence relevant to Extended Producer Responsibility (EPR) frameworks and waste management policy, with particular attention to the role of national producers in addressing plastic pollution.

## Methods

### Litter collection and clean-up methodology

The collection of anthropogenic litter, the involvement of citizen scientists and volunteers, and methods of waste auditing are described in our previous publication (Mayoma et al. 2025). Here, the salient aspects are briefly reiterated. The Clean Shores, Great Lakes clean-up campaign was conducted in two phases from February to April 2023 (35 sites) and August to September 2023 (34 sites) along the Tanzanian shores of the African Great Lakes, Victoria (48 sites), Tanganyika (9 sites), and Nyasa (12 sites). Clean-ups were conducted at sites that cause litter influx into the African Great Lakes such as markets, rivers, and beaches where the main activities are related to fishing (market, landing site), transportation (ferry ports), trade (general markets), and tourism. Only macro-litter (items > 2 cm) visible at the surface of the sand or shallow water was collected by hand.

Post-clean-up waste auditing protocols were conducted according to previously established methodologies (Barnardo and Ribbink 2020; Ocean Conservancy 2023). Litter collected in bags was aggregated and weighed (kg) prior to auditing. A representative random sample of at least 10% of the total litter collected was selected for waste auditing (mean ± standard deviation: 21.4 ± 10.4%; range: 10.0–66.6%). Waste-audited litter was enumerated into twelve main categories: plastic, cloth, paper, fishing gear, glass, sanitary, metal, medical, electrical, rubber, ceramic, and wood, each containing a number of subtypes (Mayoma et al. 2025). Waste audit data were recorded on hardcopy forms and subsequently digitised into Microsoft Excel. Enumerated litter counts from the audited subset were multiplied up to represent 100% of the litter collected at each site, based on the assumption that the randomly selected audited fraction was representative of the total litter assemblage. The compiled dataset for each clean-up site, together with photographic documentation of events, was uploaded to the project website (https://cleanshoresgreatlakes.norceresearch.no/).

### Waste audit and derivation of the branded plastic dataset

**Fig. 1:**
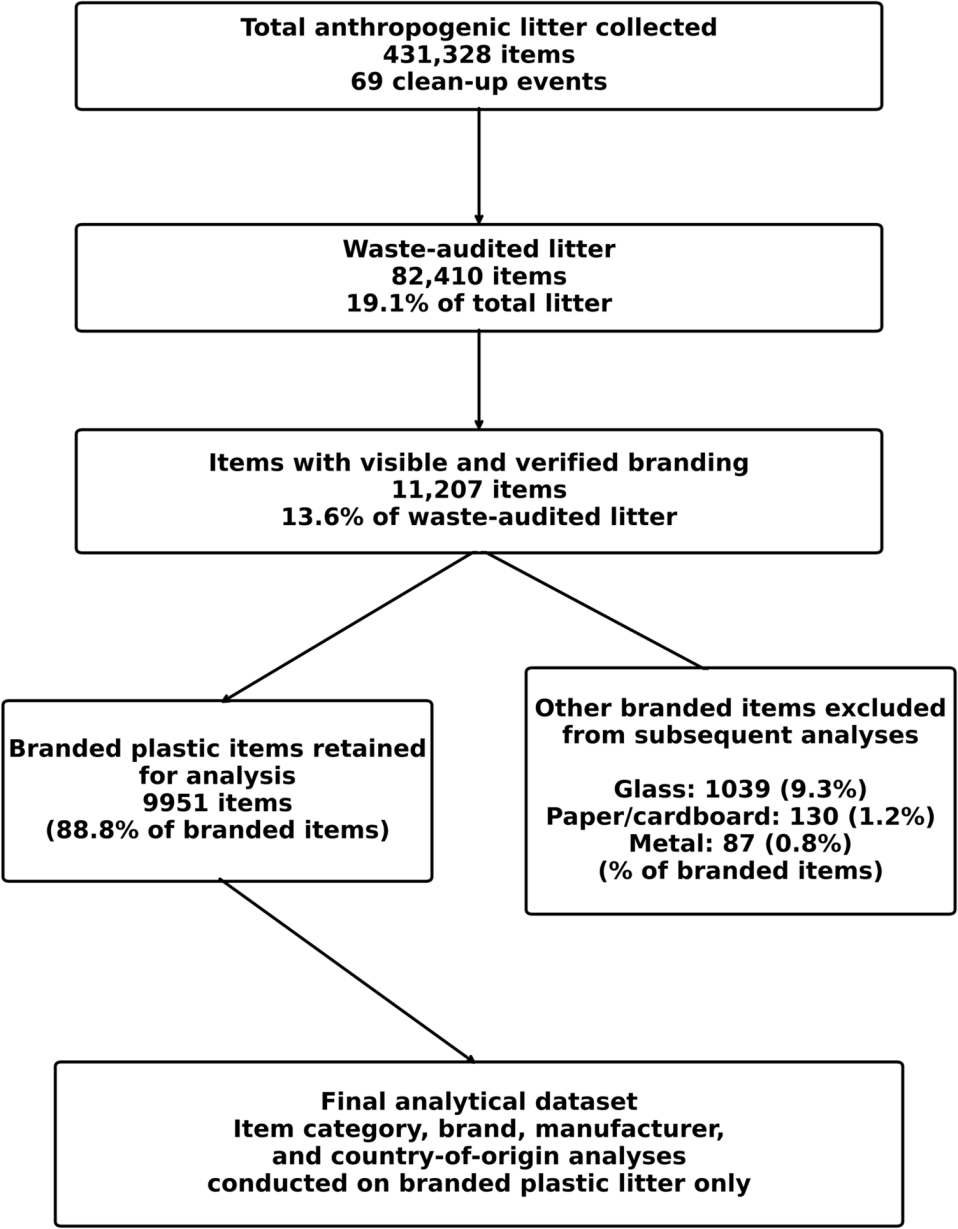
Methodological workflow showing derivation of the branded plastic litter dataset**.** Across all clean-up events 431,328 anthropogenic litter items were collected, of which 82,410 underwent waste auditing. Visible branding was identified and verified on 11,207 waste-audited items. Plastics accounted for 9951 branded items, which constituted the final analytical dataset used for item category, brand, manufacturer, and origin analyses. Non-plastic branded items (glass, paper/cardboard and metal) were recorded but excluded from subsequent analyses

Across all sixty-nine clean-up events, 82,410 pieces of anthropogenic litter underwent waste auditing, representing 19.1% of the total 431,328 items collected by the Clean Shores, Great Lakes project. Within the waste-audited subset, branding was identifiable and verified on 11,207 items (13.6%). Plastics accounted for the majority of the branded litter items (9951 items, 88.8%) followed by glass (1039 items, 9.3%), paper/cardboard (130 items, 1.2%) and metal (87 items, 0.8%). Thus, the present study focuses exclusively on the plastic litter fraction because plastics represented the dominant category of branded litter and the overwhelming majority of identifiable branded items. Analyses of item categories, brands, manufacturers, and country of origin were restricted to the 9951 branded plastic items comprising the final dataset (Fig. 1, also see Supplementary Table S1. Full dataset of Clean Shores Great Lakes Brand Audit).

### Brand audit and product attribution

Branded plastic items were specified to waste category, item category, brand name, manufacturer, and country of origin. Relevant plastic item categories included food-related items such as beverage bottles, food containers, cups and plates, and food wrappers, as well as soft plastics typically used for the packaging of non-food items (e.g. detergents and tissues). The brand name was defined as the product name visible on the litter item, while the manufacturer refers to the corporate entity responsible for producing or owning the product brand (e.g. The Coca-Cola Company or PepsiCo), rather than the polymer producer (Okuku et al. 2024; Cowger et al. 2024). Manufacturers’ names are often not available on products, and one company can be responsible for numerous brand names (Cowger et al. 2024). Where possible manufacturers were identified directly from packaging information. Where manufacturer information was unclear, targeted online searches were conducted using combinations of visible brand names, product names and manufacturer names to verify product ownership and country of origin. Verification was based primarily on official company websites and product distributors (Garcés-Ordóñez et al. 2025). In cases involving licensing, bottling agreements, or changes in ownership, attribution was assigned to the company responsible for product manufacture or distribution within the Tanzanian market at the time of sampling. Country of origin was classified as national (Tanzanian), regional (neighbouring East African countries sharing a land or Great Lakes border, such as Kenya), or international (originating from outside the East African region). Country of origin classifications were verified through online searches where required.

### Data cleaning and validation

Brand audit data present specific challenges due to both inconsistencies in data entry and the need to link branded items accurately to their manufacturer. Following initial curation of the dataset in Microsoft Excel, a series of manual validation steps were undertaken to harmonise brand and manufacturer names. For example, variations in the brand names were standardised, such as correcting “Coca Cola”, “Coca cola”, and “Coca-cola” to the official brand name “Coca-Cola”. Similarly, multiple variants of “U-Fresh Mango” (the correct brand name), including “u-Fresh Mango”, “Ufresh Mango”, and “U-fresh Mango”, were consolidated into a single standardised entry. Consistency checks were also applied to manufacturer names (e.g. The Coca-Cola Company), and online searches were again conducted where necessary to verify that products had been attributed to the correct manufacturer and associated with the appropriate country of origin.

Numerical validation of the brand audit dataset was subsequently conducted by importing the Excel spreadsheets into R (version 4.4.1) using RStudio (version 2026.01.0, Build 392). Data were checked for missing values, zero counts, and categorical inconsistencies, which were corrected prior to analysis. Item counts were then aggregated by waste category, item category, brand, manufacturer, and origin. Absolute counts, percentage contributions, and cumulative contributions were calculated to characterise the distribution and concentration of branded plastic litter across item categories, manufacturers, and country of origin. The final brand audit dataset was summarised using total recorded items, waste-audited items, and brand-audited items, and expressed as proportions of the relevant totals.

### Data analysis and visualisation

All visualisation was done in R, using tidyverse and the ggplot2 package. Analyses are descriptive in nature and focused on observed distributions and concentration patterns within the compiled branded plastic litter dataset. Results were presented using bar charts, pie charts, and cumulative contribution plots. Bar charts show absolute item counts ordered by descending frequency, while pie charts summarise relative contributions of the most abundant categories or companies, with less frequent entries combined as “other” where appropriate.

## Results and discussion

### Plastic item categories and abundant product brands

**Fig. 2:**
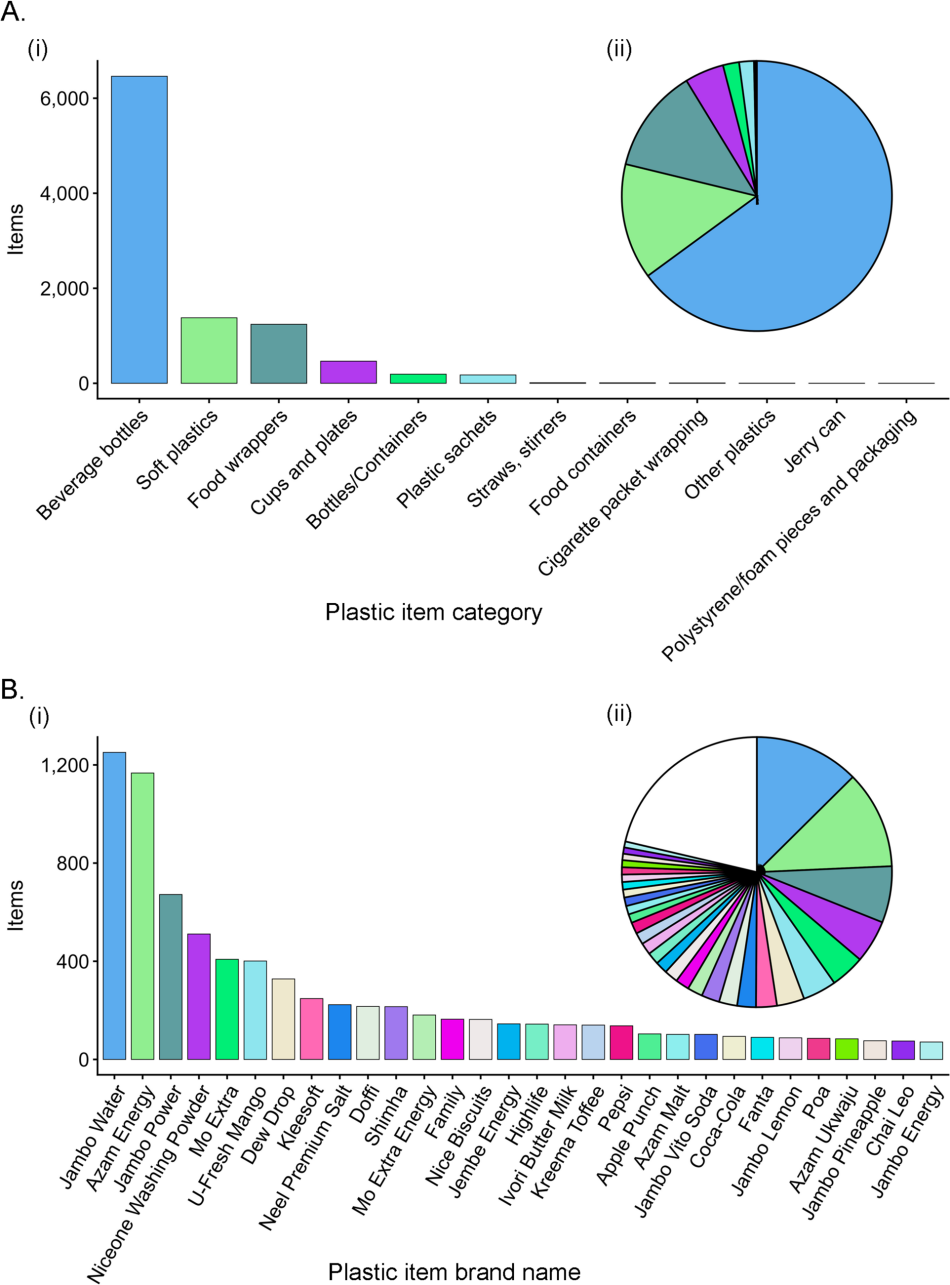
**A** Distribution of plastic litter by item category showing absolute item counts (i) and relative contributions of each category (ii). **B** Distribution of branded plastic litter by product brand, showing absolute item counts for the thirty most frequently recorded brands (i) and their relative contributions (ii), with the white pie segment representing the combined contribution of all remaining brands

Branded plastic litter (9951 items in total) was dominated by single-use “fast-moving consumer goods”. Plastic beverage bottles (6462 items, 64.9%) were the most abundant item category, followed by soft packaging (1379 items, 13.9%) and food wrappers (1242, 12.5%). All remaining plastic item categories individually accounted for less than 5% of branded plastic litter and collectively contributed less than 9% of items (Fig. 2A). The dominance of branded plastic beverage bottles is consistent with patterns reported previously from the Clean Shores, Great Lakes dataset, where plastic bottles accounted for approximately 20% of all anthropogenic litter (Mayoma et al. 2025). However, with the exclusion of unbranded items, most notably weathered and fragmented plastic bags, which also constituted around 20% of total litter in the earlier study, plastic beverage bottles were proportionally more important within the branded litter subset. The prevalence of single-use items, particularly those used in food and beverage packaging, is consistent with the literature from the region (Ngupula et al. 2014; Mayoma et al. 2019) and more broadly (Baxter et al. 2022; Nguyen et al. 2022; McGoran et al. 2023; Ocean Conservancy 2023) across both freshwater and marine environments, reflecting patterns of human consumption that appear to be universal.

**Fig. 3:**
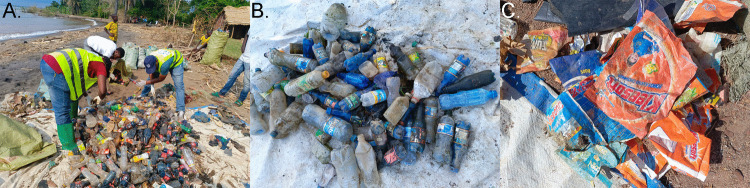
Examples of brand auditing conducted during citizen science clean-ups. **A** Citizen scientists conducting brand audits during shoreline clean-up activities. Collection of Jambo Water plastic bottles (**B**) and Niceone washing powder packaging (**C**), shown as examples of frequently recorded branded plastic items

Analysis of plastic item brand names further supported the dominance of plastic beverage bottles as the main branded litter type and showed that a relatively small number of brands accounted for a large proportion of branded plastics. Overall, 292 individual brands were recorded during brand auditing. Nineteen of the thirty most abundant branded items (Fig. 2B) were plastic beverage bottles, with the remaining items largely comprising food or product packaging (e.g. personal care or cleaning products). The three most abundant branded items recorded were beverage bottles, namely Jambo Water (1251 items, 12.6%), Azam Energy (1167 items, 11.7%), and Jambo Power (672 items, 6.8%). The fourth most abundant branded item was packaging from Niceone washing powder (511 items, 5.1%) (Fig. 2B; Fig. 3). The top eight brands together contributed 50.1% of all branded plastic litter, all of which were domestically produced. International brands, Pepsi (137 items, 1.4%), Coca-Cola (94 items, 0.9%), and Fanta (90 items, 0.9%), were present within the top thirty brands but contributed relatively small proportions to the branded plastic litter. These differences are explored further by aggregating brands to their manufacturer in the following section.

### Manufacturer attribution and corporate contribution

**Fig. 4:**
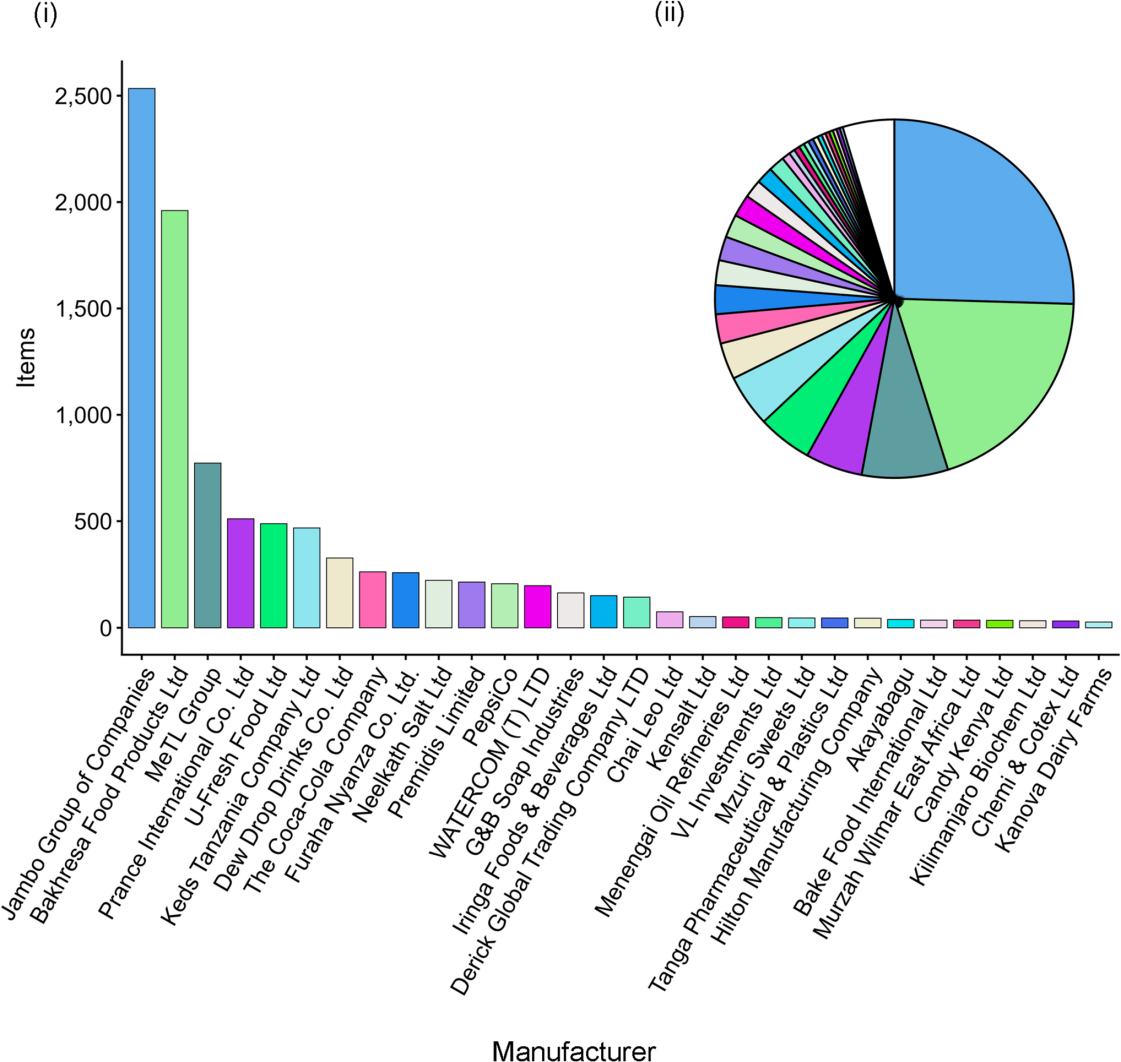
Distribution of branded plastic litter aggregated to the manufacturer level, showing absolute item counts for individual manufacturers ordered by descending frequency (**i**) and their relative contributions (**ii**), with the white pie segment representing the combined contribution of all remaining companies

In total, 148 individual manufacturers were identified within the branded plastic litter dataset. The three highest-ranked manufacturers were the Jambo Group of Companies, Bakhresa Food Products Ltd and MeTL Group (Fig. 4). Jambo Group of Companies alone accounted for over a quarter of all brand-audited plastic litter (25.5%, 2534 out of 9951 branded plastic items) and is responsible for drinks brands such as Jambo Water (most frequently recorded branded item, see Fig. 2 for common brands), Jambo Power (third most recorded item), Jambo Vito Soda and Jambo Lemon, as well as assorted food products. Overall, Jambo Group of Companies had 27 brands among the 292 brands recorded during auditing. Bakhresa Food Products Ltd (1960 items, 19.7%) owns the Azam range of drinks including Azam Energy (second most recorded item), Azam Malt and Azam Embe, as well as other beverages and food products represented within the top thirty branded items.. In total, 25 Bakhresa Food Products Ltd-owned brands were recorded during brand auditing. The third most frequently identified manufacturer was MeTL Group (773 items, 7.8%), to which the Mo drinks range (Mo Extra (fifth most common brand), Mo Extra Energy and Mo Malt) is attributable, in addition to wrapped foods and detergent products. Ten brands from MeTL Group were identified during brand auditing of plastic litter. It is also worth noting that the fourth-ranked manufacturer, Prance International Trade Co. Ltd (511 items, 5.1%), a registered company based in Dar es Salaam, was responsible for a single brand, namely Niceone Washing Powder (fourth most commonly found brand), while the fifth-ranked manufacturer, U-Fresh Food Ltd (488 items, 4.9%), produces a range of U-Fresh branded cupped drinks, most notably U-Fresh Mango (sixth most commonly found brand). The top three manufacturers together accounted for over 50% of the branded plastic litter (52.9%), while the combined contribution of the remaining 145 manufacturers was less than half. All remaining manufacturers except Prance International Trade Co. Ltd individually accounted for less than 5% of total branded plastic litter.

**Fig. 5:**
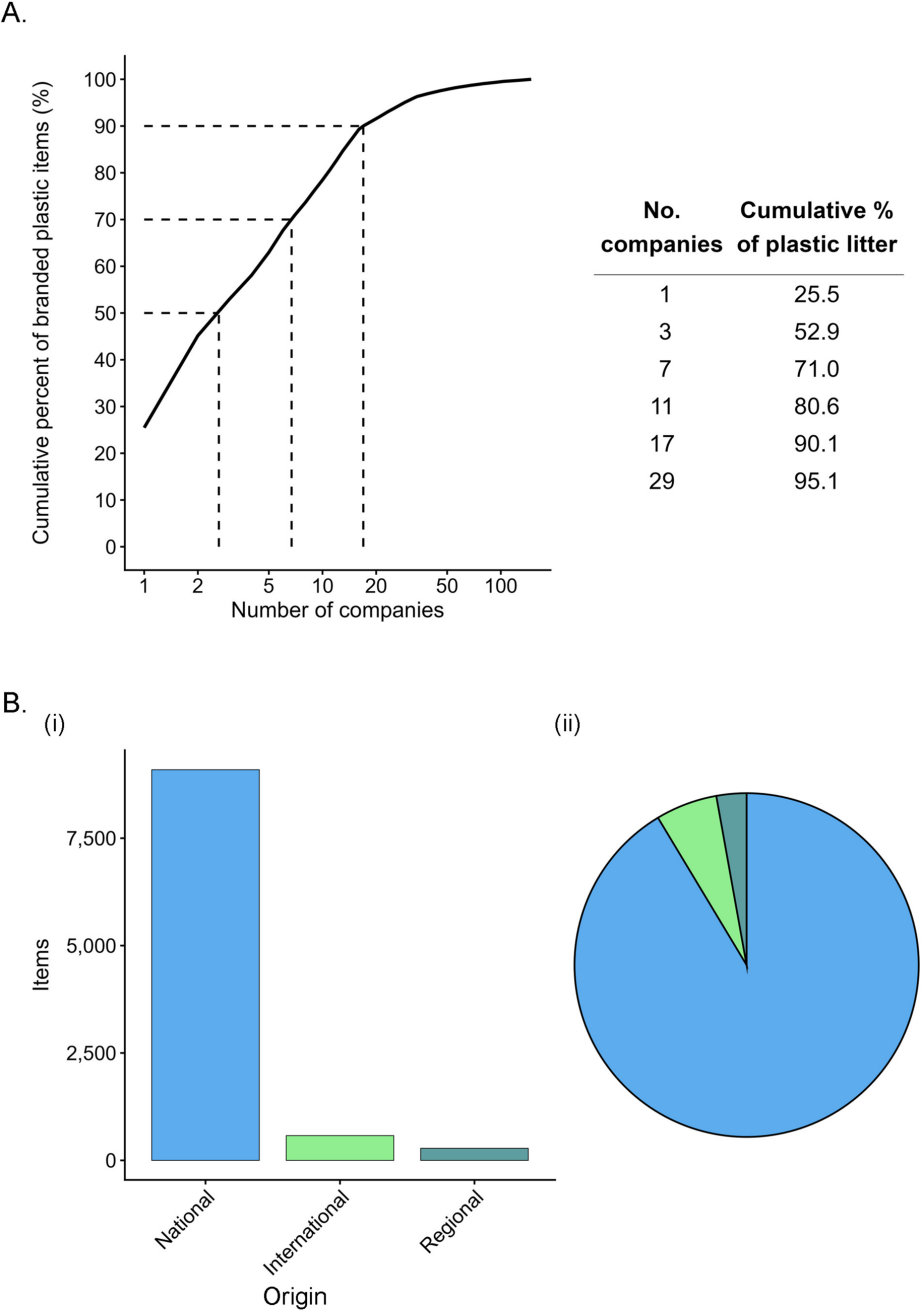
**A** Cumulative contribution (in %) of manufacturers to branded plastic litter, with the x-axis (number of companies) shown on a log10 scale. Dashed lines indicate key thresholds, illustrating the number of companies required to account for 50%, 70%, and 90% of branded plastic litter with a range of relevant corresponding values summarised in the accompanying table. **B** Origin of branded plastic litter aggregated by manufacturer as being national, regional, or international, showing absolute item counts by origin category (i) and relative contributions (ii)

The concentration of branded plastic litter is further illustrated by the cumulative contribution curve (Fig. 5A), which demonstrates the disproportionate contribution of a small subset of manufacturers. The top three manufacturers together contributed 52.9% of branded plastic litter, while the seven most abundant manufacturers accounted for 71.0%. Expanding further, 17 manufacturers explained 90.1% of branded plastic litter and only 29 manufacturers were sufficient to account for 95.1% of the total. Rather than responsibility being broadly distributed across hundreds of producers, the cumulative contribution analysis demonstrates that branded plastic pollution within the Tanzanian African Great Lakes is highly concentrated among a relatively small number of companies. This pronounced concentration has important implications for producer responsibility frameworks because it suggests that targeted engagement with a limited number of manufacturers could potentially address a substantial proportion of identifiable branded plastic pollution. A similar power-law pattern has been reported at the global level, where in absolute terms, 56 companies were required to account for 50% of branded plastic litter worldwide (Cowger et al. 2024). The higher concentration of manufacturer attribution observed in the present study suggests that, within this set of clean-up locations, corporate responsibility for branded plastic waste is even more narrowly distributed.

At the global scale, large multinational companies are predominantly accountable, with The Coca-Cola Company (11%), PepsiCo (5%), Nestlé (3%), Danone (3%), and Altria (2%) the top five manufacturers worldwide (Cowger et al. 2024). The dominance of these companies is often also seen in different parts of the world (Burt et al. 2020; Youngblood et al. 2022; Savage et al. 2024). However, in the present study, Tanzanian manufacturers accounted for the vast majority of branded plastic items (91.4%), while international (5.8%) and regional (2.8%) manufacturers comprised comparatively minor proportions of the total (Fig. 5B). International manufacturers were present in the branded plastic litter, notably The Coca-Cola Company (263 items, 2.6%) and PepsiCo (207 items, 2.1%) as the eighth- and twelfth-ranked manufacturers but represented only a small fraction of local litter despite their prominence in global assessments. Regional brands and manufacturers, defined as being from neighbouring East African countries sharing a land or Great Lakes border, were exclusively of Kenyan origin and represented an assorted collection of products. Supporting our findings in the Great Lakes region, brand audits in Kenyan Rift Valley lakes revealed that 98.6% and 99.2% of branded litter in Lakes Baringo and Naivasha beaches, respectively, were of national origin (Okuku et al. 2024), highlighting the importance of domestic market share within inland locations.

Our findings from Tanzanian shoreline of the African Great Lakes demonstrate that branded plastic litter is highly concentrated among a small number of nationally based manufacturers. This has important implications for corporate responsibility frameworks, particularly EPR where clearly identifiable and domestic stakeholders may facilitate targeted regulatory and waste management interventions.

### Implications for extended producer responsibility and waste management

EPR has emerged as an important policy instrument in ongoing negotiations toward a global legally binding instrument on plastic pollution, although generally positioned in the waste management section of the value chain (Dreyer et al. 2024). This reflects the nature of EPR as a policy approach, in which it is considered an operational extension of the Polluter Pays Principle, whereby producers leverage corporate resources to take responsibility for their product at end of life (Raubenheimer and Urho 2020; Dreyer et al. 2024). Thus, typical EPR frameworks assign responsibility to manufacturers for the post-consumer phase of their products’ lifecycle, including collection, recycling, and waste management. However, it is important to distinguish between product manufacturers and upstream polymer producers who produce plastic raw materials. Companies such as Exxon Mobil and Dow that produce plastics are not directly linked to plastic litter through brand audits (Baxter et al. 2022). Given that brand audits attribute litter to the manufacturers of the consumer products, our study provides a clear demonstration of the potential applicability of EPR frameworks within the Tanzanian boundaries of the African Great Lakes, where branded plastic litter is traceable to a limited number of identifiable manufacturers.

The corporate concentration observed in this study presents a clear governance opportunity. With three nationally based manufacturers accounting for over half of branded plastic litter, seven nationally based manufacturers accounting for 71% and less than 30 companies in total accounting for 95% of the total, responsibility is neither diffuse nor anonymous. Instead, it is concentrated among a relatively small number of nationally based companies operating within the Tanzanian market. This increases the feasibility of implementing targeted EPR mechanisms, as regulatory engagement can focus on a clearly defined group of companies within the same food and beverage sector. In practical terms, mechanisms such as bottle-return schemes, producer-financed collection systems, and contributions to waste infrastructure, as implemented elsewhere (Mwanza et al. 2018; Ramasubramanian et al. 2023), could be strategically directed toward the most significant contributors. Whether voluntary or mandated by national government or a global agreement (Cowger et al. 2024), action from these manufacturers could make a significant impact in substantially reducing environmental leakage. Our specific recommendation would be for those top three manufacturers, all of whom own drinks brands that were well represented in litter, to collectively implement return schemes or collection points that address plastic bottles. Given the dominance of beverage-related brands within the branded plastic dataset, such measures could potentially address a substantial proportion of branded plastic litter and contribute to reductions in overall anthropogenic litter within the Tanzanian African Great Lakes. However, implementation feasibility depends on broader considerations including collection logistics, informal waste sectors, market distribution systems, and national policy capacity.

At the same time, it must be recognised that consumers, particularly in Africa, often lack viable alternatives to environmental leakage. Proper and efficient waste collection and infrastructure are absent from much of Africa (Adebiyi-Abiola et al. 2019); thus, the persistence of plastic litter witnessed during clean-ups reflects broader structural limitations in waste management systems. A coalition of national manufacturers could decide together to strengthen the domestic waste management infrastructure around the Great Lakes in an act of Corporate Social Responsibility. Whilst international manufacturers also subscribe to Corporate Social Responsibility rhetoric, they may simultaneously employ tactics that hinder real progress, borrowing from the playbook of corporate climate delay tactics (Vandenberg 2024). In the national context, coordinated and transparent action to finance waste infrastructure and implement EPR mechanisms would send a clear and credible signal of commitment to pollution mitigation. Whilst community-led clean-ups have an important role, without parallel producer responsibility measures and infrastructure development, the burden of waste management continues to fall disproportionately on local communities.

## Conclusions

Branded plastic litter in the Tanzanian African Great Lakes was highly concentrated among a small number of nationally based manufacturers, with beverage bottles representing the dominant item category. The findings demonstrate that responsibility for branded plastic pollution is traceable to very few identifiable corporate actors, providing a strong basis for targeted Extended Producer Responsibility frameworks and producer-led waste management interventions. Thus, we can draw a straight line from visible litter to corporate responsibility. Identified manufacturers should involve themselves in the provision of waste management infrastructure to prevent environmental leakage. Without such engagement, the burden of plastic pollution in the African Great Lakes falls disproportionately on community initiatives, while the impacts on biota, ecosystems and increasingly human health continue to rise.

## Supplementary Information

Below is the link to the electronic supplementary material.

Supplementary Material File 1 (XLSX 24.7 KB)

## Data Availability

All data generated or analyzed during this study are included in this published article and its supplementary information file (Table S1. Full dataset of Clean Shores Great Lakes Brand Audit).
